# Stand Structure and Type Drive Productivity in Chinese Fir Forests: Comparison of Pure and Mixed Stands

**DOI:** 10.1002/ece3.72750

**Published:** 2026-01-11

**Authors:** Yang Guo, Xunzhi Ouyang, Ping Pan, Jianfeng Li, Jun Liu

**Affiliations:** ^1^ Key Laboratory of National Forestry and Grassland Administration for the Protection and Restoration of Forest Ecosystem in Poyang Lake Basin, College of Forestry Jiangxi Agricultural University Nanchang People's Republic of China

**Keywords:** *Cunninghamia lanceolata*, pure and mixed stand, stand productivity, stand structure, structural equation modeling

## Abstract

Understanding the main factors that influence productivity across stand types is essential for effective management strategies. Chinese fir ( *Cunninghamia lanceolata* ) is a key afforestation species in subtropical China. However, comparative studies on productivity differences and the underlying mechanisms among different Chinese fir stand types remain scarce. In the present study, using second‐class forest resource survey data from Ganzhou, southern China, we classified plots dominated by Chinese fir into three stand types: pure Chinese fir (541 plots), Chinese fir–broadleaf mixed (351 plots), and Chinese fir–conifer mixed stands (232 plots). We then assessed productivity differences among stand types and applied the Boruta feature selection and Random Forest modeling to identify the main influencing factors, followed by structural equation modeling (SEM) to evaluate their direct and indirect effects. The results indicated that stand productivity differed significantly among stand types, with stand density, dominant diameter, stand age, Gini coefficient, and mean tree height being the most influential factors. The productivity of pure stands was more sensitive to climatic and site conditions, whereas mixed stands were more strongly influenced by structural heterogeneity. These findings highlight the critical role of stand structure in regulating productivity and suggest that enhancing density regulation in all stands and promoting structural heterogeneity in mixed stands can improve productivity and stability, with mixed‐stand management offering greater potential for long‐term productivity gains and resilience to climate change.

## Graphical abstract

We compared stand productivity and its key drivers across pure and mixed Chinese fir ( *Cunninghamia lanceolata* ) forests in subtropical China. Stand density, dominant diameter, stand age, Gini coefficient, and mean tree height were the main factors affecting productivity, with pure stands showing significantly higher productivity but also greater climate sensitivity. Our findings suggest that converting pure to mixed stands can enhance ecosystem stability and long‐term productivity.

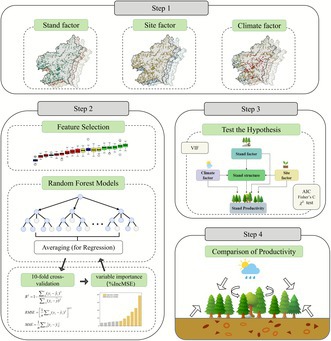

## 1 Introduction

Improving vegetation productivity is a major strategy to enhance terrestrial carbon uptake and can play a crucial role in mitigating climate change and maintaining ecological balance (Liu et al. 2025). Forest productivity is a critical indicator of the production capacity and ecological functions of forests. Diversity–productivity relationships (DPRs) suggest that species diversity and stand structural complexity can significantly enhance forest productivity (Beugnon et al. 2025). Analyses of global forest plots (Liang et al. 2016; Feng et al. 2022) have demonstrated that mixed forests generally have higher productivity than monocultures, primarily owing to interspecific complementarity and compensatory effects. Mixed stands are also widely recognized for providing stronger ecological functions and greater resistance to disturbances than monocultures (Wang et al. 2025). However, mixed forests are not always superior to monocultures; their productivity and ecosystem services do not necessarily exceed those of pure stands dominated by native species (Li et al. 2024). Even within the same region, outcomes vary among forest types. For example, along the Iberian Peninsula, the productivity of mixtures of five pine species ( *Pinus sylvestris* , *Pinus pinea* , *Pinus halepensis* , *Pinus nigra* , and *Pinus pinaster* ) is reportedly lower than that of monospecific stands (Aguirre et al. 2019). In contrast, in another region of Europe, mixed stands of *Pinus sylvestris* and oak exhibit higher productivity than monocultures (Pretzsch et al. 2020). Therefore, the productivity of mixed versus pure stands can considerably fluctuate across contexts (Pretzsch et al. 2010). While these differences may arise from variations in tree species composition, they may also be related to the main factors influencing productivity across stand types.

Numerous studies have demonstrated that mixed‐species forests generally exhibit greater productivity than monocultures; however, these findings often vary depending on the specific species combinations and environmental conditions involved (Forrester and Bauhus 2016). Forest stand productivity is influenced by several factors, including climate (Xi et al. 2024), site condition (Li, Wang, et al. 2023; Toïgo et al. 2015), and stand factors (Pretzsch et al. 2024). Moreover, the outcomes are not always consistent across different species combinations and environmental conditions. In temperate forests, the productivity of mixed stands is mainly driven by species composition and site conditions (Toïgo et al. 2015), whereas in subtropical forests it is influenced by species diversity and stand structure (Ren et al. 2021). Increasing evidence has indicated that an appropriate stand structure not only alleviates environmental stress and reduces individual competitionbut also determines the degree to which species complementarity enhances productivity in mixed stands (Condés et al. 2023; Zhang et al. 2024). Even in pure stands, enhancing stand structural heterogeneity can substantially increase productivity (Pretzsch et al. 2024). Stand structural heterogeneity can directly regulate resource allocation and growth dominance, thereby influencing productivity. Moreover, it can act as a mediating variable that transmits the effects of other stand and environmental factors (Gao et al. 2021; Wang et al. 2024). Notably, the optimization of stand structure can be achieved through various methods, such as the presence of dominant trees (Shi et al. 2025), appropriate density regulation (Oliveira et al. 2024), and improved age‐class structure (Pretzsch et al. 2024), thus maintaining high productivity across different stages of stand development. This also provides theoretical support for exploring the relationship between structure and function.

Chinese fir ( *Cunninghamia lanceolata* (Lamb.) Hook.) is an endemic species that is intensively cultivated in the subtropical regions of southern China. The species is valued for its high economic return and superior wood quality and is a primary species used in commercial plantation forestry. However, owing to the combined effects of climate, site conditions, and simplified stand structure, some Chinese fir plantations have experienced challenges related to low productivity and poor efficiency (Farooq et al. 2019). Given the potential advantages of mixed Chinese fir forests in terms of ecological stability and multifunctionality, promoting the transition from pure coniferous plantations to more complex and stable mixed‐species forest management has become an important direction for the ecological transformation of plantation forests (Wang, Dong, and Liu 2022). Recent studies on Chinese fir forests have mainly examined the biodiversity–ecosystem function relationship, generally demonstrating that tree species diversity positively affects productivity (Yang et al. 2023; Mo et al. 2025). However, most existing studies on Chinese fir have relied on a priori assumptions for factor selection or interpretation (Liu et al. 2024; Hu et al. 2025), potentially overlooking interactions among multiple factors. In addition, systematic comparisons of productivity differences and driving mechanisms across stand types under the same regional conditions are lacking. These knowledge gaps limit our understanding of productivity improvement in Chinese fir forests, highlighting the need for large‐scale plot data and integrated analytical approaches.

**FIGURE 1:**
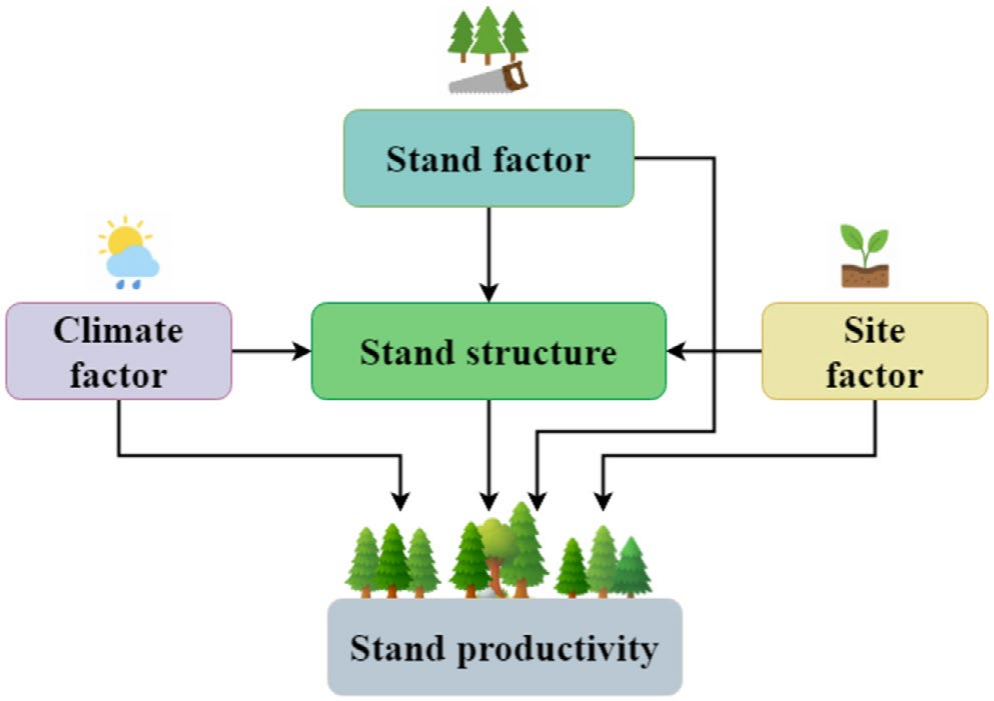
Conceptual path model of climate, site, and stand factors influencing productivity through stand structure.

Therefore, in the present study, we focused on Ganzhou City, Jiangxi Province, a major production area of Chinese fir. Plots dominated by Chinese fir were classified into Chinese pure, Chinese fir–conifer mixed, and Chinese fir–broadleaf mixed stands. We assessed stand productivity as the mean annual increment of stand volume (Husch et al. 2003). Using Random Forest (RF) for factor selection and structural equation modeling (SEM) for path analysis (Li, Wu, et al. 2023; Luo et al. 2023), we examined how climate, site, and stand factors influence productivity through stand structure. We hypothesized that (1) productivity significantly differs among stand types; and (2) climate, site, and stand factors affect productivity directly or indirectly via stand structure (Figure 1), with varying effects across stand types.

## 2 Materials and Methods

### 2.1 Study Area

The study area was located in Ganzhou City, Jiangxi Province (24°29′–27°09′ N, 113°54′–116°38′ E), southeastern China. Its topographic features include low mountains and hills, with an elevation of 82–2061 m. The region has a subtropical monsoon climate, with an annual average temperature and precipitation of 19.1°C–20.8°C and 1580 mm and a frost‐free period of 288 days. The forest coverage rate is 76.2%, and the zonal vegetation consists of subtropical coniferous and evergreen broadleaf forests. The main tree species include Chinese fir, *Pinus massoniana* , *Schima superba*, and *Castanopsis fargesii*. Chinese fir forests in the study area cover 704,000 ha—26.2% of the total forested area.

### 2.2 Data Sources

#### 2.2.1 Inventory of Plots

**FIGURE 2:**
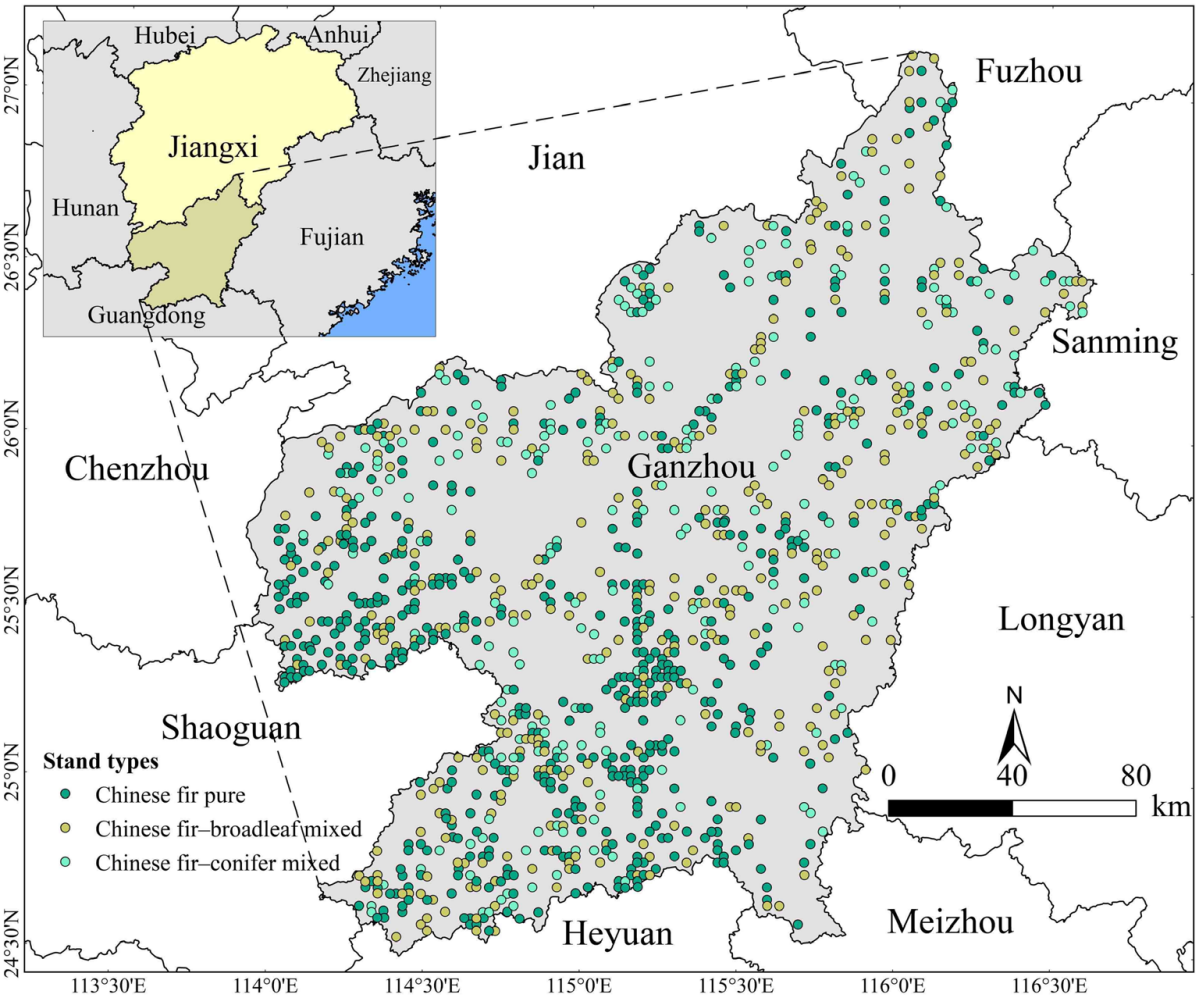
Location of the study area and distribution of Chinese fir pure (CF), Chinese fir–broadleaf mixed (CFB), and Chinese fir–conifer mixed (CFC) plots.

Plot data were obtained from the 2019 forest management inventory (second‐class forest resource survey) database for Ganzhou. Each plot covers an area of 0.08 ha (28.28 × 28.28 m). The geographic coordinates were based on the southwest corner of each plot. The diameter at breast height (DBH) (i.e., 1.3 m) was recorded individually for all trees with a DBH of ≥ 5 cm. In addition, site factors, such as slope aspect and slope gradient, and stand factors, including stand age and mean tree height, were recorded. Sample plots where in Chinese fir was the dominant species (accounting for ≥ 65% of stand volume) were selected from the plot database and classified into three stand types: pure Chinese fir stands (CF), where in Chinese fir accounted for ≥ 90% of the stand volume; and mixed stands, where in Chinese fir accounted for between 65% and 90% of the stand volume. Based on the characteristics of the main accompanying species, mixed stands were further divided into Chinese fir–broadleaf mixed stands (CFB) and Chinese fir–conifer mixed stands (CFC). The dominant accompanying species in Chinese fir–broadleaf mixed stands included *Schima superba*, *Castanopsis fargesii*, *Liquidambar formosana*, *Camphora officinarum*, and *Alniphyllum fortunei*. Those in Chinese fir–conifer mixed stands included *P. massoniana* and *P. elliottii* . Based on the plot database, we selected 545, 351, and 232 pure Chinese fir, Chinese fir–broadleaf mixed, and Chinese fir–conifer mixed plots, respectively. Plot distribution is depicted in Figure 2.

#### 2.2.2 Climate Data

For each plot, climate data were generated from longitude, latitude, and elevation using ClimateAP v3.10 (Wang et al. 2017), released in 2023 with data spanning 1901–2021. The climate variables used in the modeling were averaged over the stand age and year of survey for each plot. The selected climate factors included mean annual precipitation and temperature, mean temperatures of the coldest and warmest months, and relative humidity.

#### 2.2.3 Soil Data

Soil data were acquired from the global digital soil mapping system (https://www.soilgrids.org/) (Hengl et al. 2017), with a resolution of 250 × 250 m. Soil organic carbon and total nitrogen, which are indicators of soil fertility, and bulk density, which reflects soil structure, were selected as explanatory variables influencing Chinese fir productivity. Based on the geographic coordinates and soil thickness in each plot, soil data at corresponding depths were extracted from the relevant raster maps, and the average values were calculated.

### 2.3 Methods

#### 2.3.1 Stand Productivity Calculation

The stand volume mean annual increment (m^3^·ha^−1^·year^−1^) was used to measure stand productivity (Liang et al. 2016; Yan et al. 2023). This indicator not only integrates the cumulative effects of stand growth over the entire life cycle (Pretzsch et al. 2023) but also exhibits good sensitivity to climate and site variations (Tian et al. 2024; de Esther Lima Costa et al. 2020). It was calculated as follows:

(1) $$\mathrm{VAI}=\frac{V}{\mathrm{AG}}$$

where *VAI* is stand productivity (m^3^·ha^−1^·year^−1^), *V* is stand volume (m^3^·ha^−1^), and AG is stand age (years). *V* was calculated as the sum of single‐tree volumes, with those for each species obtained from one‐way volume tables for the Gannan region of Jiangxi Province.

#### 2.3.2 Selection of Factors

Based on the plot data and related studies (Matsuo et al. 2025; Wang et al. 2021), we chose 20 site, stand, and climate variables that can affect Chinese fir stands were selected (Table 1). Qualitative site factors, including slope position and aspect, were numerically coded as follows: slope position as 1 (summit/upper slope), 2 (midslope), 3 (footslope/valley/flat land), and 4 (entire slope); aspect as 1 (shady), 2 (semi‐shady), 3 (semi‐sunny), 4 (sunny), and 5 (no aspect).

**TABLE 1:** Statistical analysis of the influencing factors.

Factor	Variable	Stand type	Minimum	Maximum	Mean	SD
Site	Elevation (m)	CF	47	1150	392	142
		CFB	120	1070	398	150
		CFC	127	950	351	136
	Soil humus thickness (cm)	CF	0	30	7.44	4.91
		CFB	0	30	8.38	5.39
		CFC	0	24	7.11	4.70
	Litter leaf thickness (cm)	CF	0	20	4.52	3.31
		CFB	0	20	4.74	3.20
		CFC	0	20	4.74	3.66
	Soil thickness (cm)	CF	25	120	79.71	13.59
		CFB	20	110	77.29	13.46
		CFC	20	100	76.03	13.76
	Slope gradient (°)	CF	0	51	27	8
		CFB	3	49	27	8
		CFC	0	46	25	8
	Soil organic carbon (g·kg^−1^)	CF	9.28	31.08	17.96	3.33
		CFB	8.76	41.95	18.15	3.72
		CFC	10.28	41.90	17.00	3.92
	Total nitrogen (g·kg^−1^)	CF	0.96	2.67	1.41	0.21
		CFB	0.97	2.91	1.41	0.23
		CFC	0.92	2.61	1.34	0.23
	Bulk density (g·cm^−3^)	CF	1.15	1.41	1.32	0.04
		CFB	1.08	1.40	1.32	0.04
		CFC	1.16	1.42	1.33	0.04
	Slope position	—	—			
	Slope aspect	—	—			
Stand	Stand age (years)	CF	3	35	12	6
		CFB	3	38	18	7
		CFC	4	36	17	7
	Dominant diameter (cm)	CF	6.5	41.8	17.5	5.8
		CFB	8.6	38.8	21.5	5.9
		CFC	7.9	40.3	20.5	6.6
	Mean tree height (m)	CF	2.3	23.3	8.2	3.0
		CFB	1.5	28.4	9.0	2.9
		CFC	2.3	17.7	7.9	2.5
	Tree number per hectare (N·ha^−1^)	CF	125	7238	1983	1180
		CFB	163	5563	1862	919
		CFC	125	4963	1687	951

	Gini coefficient	CF	0.02	0.33	0.15	0.05
		CFB	0.07	0.37	0.20	0.05
CFC		0.07	0.30	0.19	0.05
Climate	Mean annual precipitation (mm)	CF	1472	2027	1733	106
		CFB	1495	2087	1719	114
		CFC	1483	1973	1693	110
	Mean annual temperature (°C)	CF	15.9	20.9	19.5	0.7
		CFB	16.7	20.7	19.4	0.8
		CFC	17.6	20.7	19.6	0.6
	Mean coldest month temperature (°C)	CF	5.7	10.8	8.99	0.8
		CFB	6.3	10.8	9.0	0.8
		CFC	7.4	10.9	9.1	0.6
	Mean warmest month temperature (°C)	CF	24.5	30.0	27.9	0.8
		CFB	25.1	29.8	27.9	0.9
		CFC	24.9	30.1	28.2	0.8
	Relative humidity (%)	CF	67	75	72	1
		CFB	66	74	72	1
		CFC	65	75	72	1

Abbreviations: CF, Chinese fir pure stand; CFB, Chinese fir–broadleaf mixed stand; CFC, Chinese fir–conifer mixed stand.

Tree number per hectare was used as an indicator of stand density. The mean diameter of the three largest trees in each plot was used as the dominant tree diameter. The Gini coefficient was used to quantify tree size inequality within the stands (Yang et al. 2019). A higher Gini value indicates a greater degree of size inequality, reflecting increased structural heterogeneity. The Gini coefficient was calculated using the *ineq* package (Zeileis 2014) as follows:

(2) $$\text{Gini}=\frac{\sum_{i=1}^{n}\;\;\sum_{j=1}^{n}\;\;\left|d_{i}-d_{j}\right|}{2n^{2}\bar{d}}$$

where *d*_*i*_ and *d*_*j*_ are the DBH of trees *i* and *j*, respectively; *n* is the number of trees in the plot; and $\bar{d}$ represents the mean DBH (cm) of the plot.

### 2.4 Statistical Analysis

As presented in Table 2, stand age, tree number per hectare, and Gini coefficient were subdivided into three levels. Stand age was classified as stand age based on Chinese fir growth stages and subdivided the tree number per hectare and the Gini coefficient into three groups (top 20%, middle 60%, and bottom 20%) based on the total number of plots of values. The differences in stand productivity among stand types were assessed using the analysis of variance (ANOVA). Figure S1 depicts the stepwise analytical framework, where the *Boruta* R package (Kursa and Rudnicki 2010) was initially used to identify the variables highly correlated with stand productivity. Boruta is an “all‐relevant” feature selection algorithm that identifies important variables by iteratively generating shadow features using an RF model and comparing importance *Z*‐scores. Variables with scores significantly higher than those of their shadow counterparts were confirmed as reflecting relevant features, resulting in a robust set of relevant factors. The RF algorithm, with its random grouping and resampling mechanisms, ensures stability and robustness in variable importance assessment. Variables were ranked by %IncMSE and the least important ones were iteratively removed until the root mean square error (RMSE) was minimized, thus identifying the optimal subset (Luo et al. 2023). Model fitting was performed using the *Random Forest* R package (Liaw and Wiener 2002) with ntree = 1000. The mtry parameter was optimized through grid search from one to the total number of variables using the *caret* package (Kuhn 2008) with 10‐fold cross‐validation, selecting the lowest RMSE as optimal. For each variable combination, model performance for each subset was evaluated based on *R* ^2^, RMSE, and MAE.

**TABLE 2:** Group categorization based on stand age (AG), tree number per hectare (*N*), and Gini coefficient (GC) value.

Variable	Groups		
Stand age (years)	≤ 10	11–20	≥ 21
Tree number per hectare (N·ha^−1^)	≤ 950 (top 20% of *N*)	951–2700 (medial 60% of *N*)	≥ 2701 (bottom 20% of *N*)
Gini coefficient	≤ 0.13 (top 20% of GC)	0.14–0.21 (medial 60% of GC)	≥ 0.22 (bottom 20% of GC)

SEMs were constructed to clarify the direct and indirect impacts of the main influencing factors on stand productivity. While the RF model can achieve excellent predictive performance even when the variables are moderately collinear, collinearity can negatively affect the accuracy of SEMs. Therefore, the *car* package (Fox and Monette 1992) in R was used to test for multicollinearity of the influence factors; the test is passed when VIF is < 5. Piecewise SEM was employed to identify the key pathways linking stand productivity and its influencing factors, fitting each sub‐model using linear regression. Variables that significantly deviated from normality or exhibited non‐linear trends were log‐transformed. Beginning with an a priori conceptual SEM containing all hypothesized paths, model fit was assessed using Fisher's C statistic and chi‐square tests (*p* > 0.05). Non‐significant paths were then sequentially removed to derive the final optimal model based on the lowest AIC. Finally, the direct, indirect, and relative impact rates of different stand types were calculated. These analyses were conducted using the *piecewise SEM* package (Lefcheck 2016) and the *semEff* package (Murphy 2022). All data analyses and visualizations were performed using R 4.2.0 (R Core Team 2020).

## 3 Results

### 3.1 Descriptive Statistics of Stand Productivity

**FIGURE 3:**
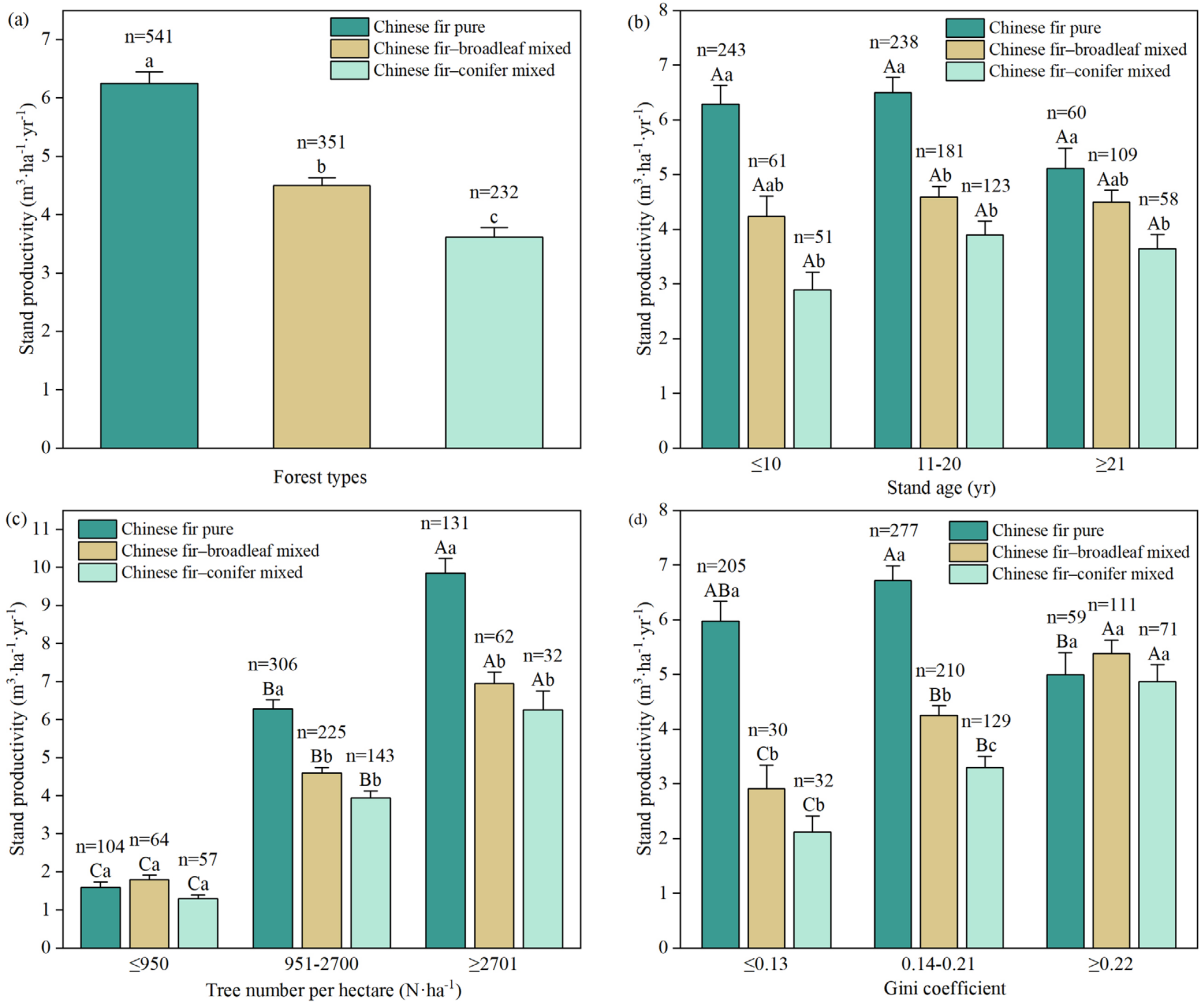
Comparison of productivity among stand types (a), stand types of various stand ages (b), stand density levels (c), and Gini coefficient levels (d). Different lowercase letters indicate significant differences among different Chinese fir stand types (*p* < 0.05); uppercase letters indicate significant differences between different groups within the same stand type (*p* < 0.05). Error bars represent standard error (SE), and *n* indicates the number of plots.

The productivity of different Chinese fir stand types is presented in Figure 3a. The average productivity of pure Chinese fir stands was 6.25 m^3^·ha^−1^·year^−1^, ranging from 0.11 to 22.01 m^3^·ha^−1^·year^−1^. The average productivity of Chinese fir–broadleaf mixed stands was 4.50 m^3^·ha^−1^·year^−1^, ranging from 0.35 to 3.19 m^3^·ha^−1^·year^−1^. For Chinese fir–conifer mixed stands, the average productivity was 3.61 m^3^·ha^−1^·year^−1^, ranging from 0.16 to 13.97 m^3^·ha^−1^·year^−1^. The differences in mean productivity among the three stand types were significant (*p* < 0.05).

The average productivity across different stand age stages ranged from 2.89 to 6.49 m^3^·ha^−1^·year^−1^ (Figure 3b). The productivity of different stand types increased initially with stand age and subsequently declined; however, the differences were not statistically significant (*p* > 0.05). The average productivity across stands at the same age stage was ranked as follows: pure Chinese fir stands > Chinese fir–broadleaf mixed stands > Chinese fir–conifer mixed stands.

The average productivity at different tree numbers per hectare ranged from 1.29 to 9.85 m^3^·ha^−1^·year^−1^ (Figure 3c). For all stand types, productivity exhibited a significant increase with increasing tree number per hectare (*p* < 0.05). At a tree number per hectare ≤ 950 N·ha^−1^, no significant differences were observed between the different stand types (*p* > 0.05). However, the productivity of pure Chinese fir stands was significantly higher than that of mixed stands (*p* < 0.05) at a tree number per hectare between 951–2700 N·ha^−1^ and ≥ 2701 N·ha^−1^.

The average productivity at different Gini coefficient levels ranged from 2.11 to 6.70 m^3^·ha^−1^·year^−1^ (Figure 3d). The productivity of pure Chinese fir stands initially increased and subsequently decreased with an increasing Gini coefficient; although the increase was not significant (*p* > 0.05), the decrease was significant (*p* < 0.05). In mixed stands, productivity consistently increased with increasing Gini coefficients. Notably, the productivity of Chinese fir–broadleaf mixed stands significantly increased at Gini coefficients ≥ 0.22 (*p* < 0.05). In Chinese fir–conifer mixed stands, productivity significantly increased across all groups (*p* < 0.05). Gini coefficients in groups were ≤ 0.13 and 0.14–0.21, and the productivity of pure Chinese fir stands was significantly higher than that of the two mixed stands (*p* < 0.05). However, no significant differences in productivity were observed among the stand types at Gini coefficients ≥ 0.22 (*p* > 0.05).

In summary, mean stand productivity followed the order of pure Chinese fir stands > Chinese fir–broadleaf mixed stands > Chinese fir–conifer mixed stands. Moreover, pure Chinese fir stands exhibited a broader range of productivity than their mixed stands. The maximum productivity for all stand types occurred in the 11–20‐year age class. Productivity increased with tree density across all stand types. A higher Gini coefficient reduced productivity in pure Chinese fir stands but enhanced it in mixed stands.

### 3.2 Factors Influencing Chinese Fir Productivity

**FIGURE 4:**
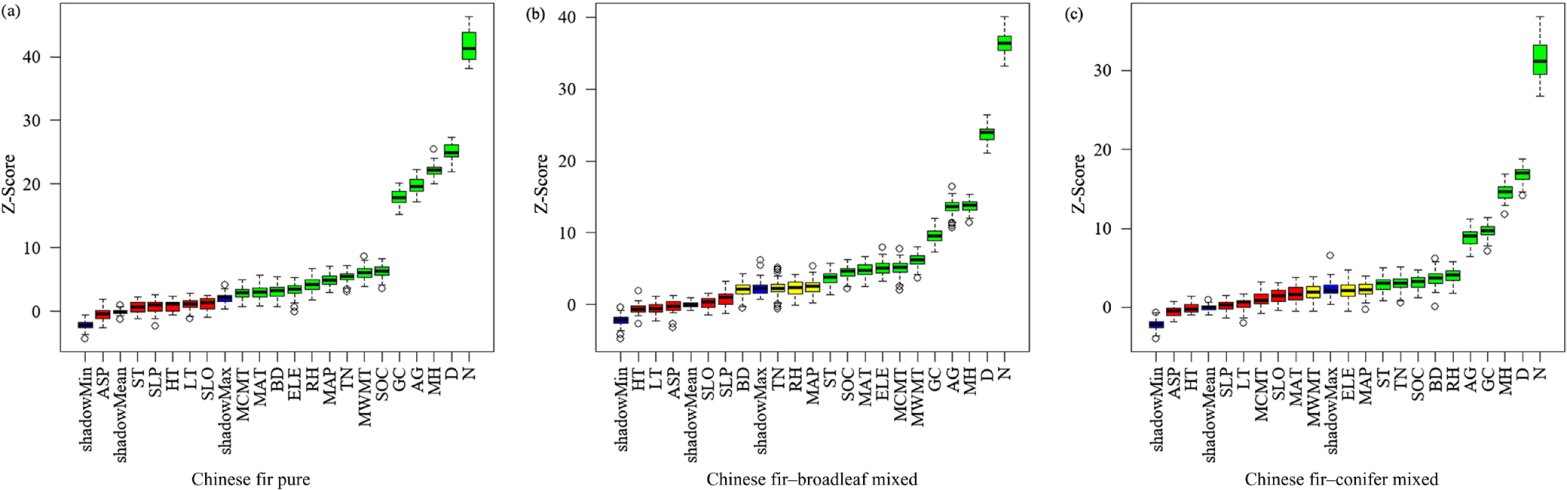
Boruta feature selection results for stand productivity across different stand types: (a) Chinese fir pure, (b) Chinese fir–broadleaf mixed, and (c) Chinese fir–conifer mixed stands. The *y*‐axis represents importance scores (*Z*‐scores), used to assess the significance of each feature relative to the shadow features (blue boxplots). Green, yellow, and red boxplots denote confirmed, tentative, and rejected features, respectively. N is tree number per hectare; D is the dominant diameter; MH is mean tree height; AG, stand age; ASP, aspect; BD, bulk density; *D*, the dominant diameter; ELE, elevation; GC, Gini coefficient; HT, soil humus thickness; LT, litter leaf thickness; MAP, annual precipitation; MAT, mean annual temperature; MCMT, mean coldest month temperature; MH, mean tree height; MWMT, mean warmest month temperature; *N*, tree number per hectare; RH, relative humidity; SLO, slope; SLP, slope position; SOC, soil organic carbon; ST, soil thickness; TN, total nitrogen.

We used the Boruta feature selection algorithm to input the same set of 20 variables (Table 1) affecting Chinese fir productivity for different stand types. The results indicated that different stand types produced distinct sets of influential factors. We identified 14, 11, and 10 important features for pure Chinese fir stands, Chinese fir–broadleaf mixed stands, and Chinese fir–conifer mixed stands, respectively (Figure 4a–c).

**FIGURE 5:**
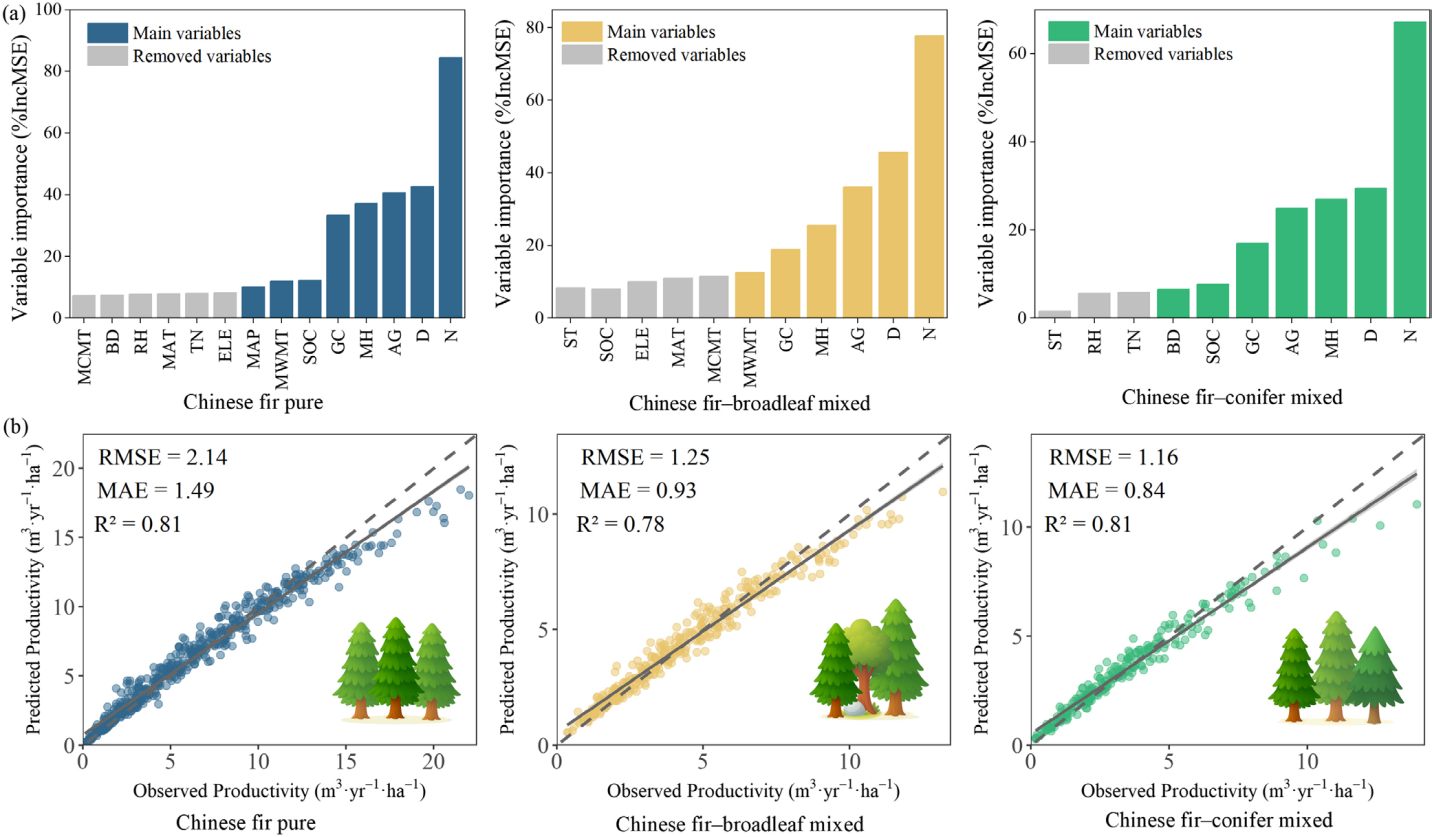
Importance ranking of the main influencing factors on productivity across different Chinese fir stand types and evaluation results of the Random Forest regression models. (a) Variable importance is expressed as %IncMSE. (b) Evaluation results of the Random Forest models based on 10‐fold cross‐validation.

We constructed RF models for pure Chinese fir, Chinese fir–broadleaf mixed, and Chinese fir–conifer mixed stands and ranked the predictors by importance (Figure 5a). To this end, we sequentially removed the least important variables until the model RMSE reached its minimum. The best‐performing models retained 8 (mtry = 7), 6 (mtry = 5), and 7 (mtry = 4) variables for the three stand types, yielding minimum RMSEs of 2.14, 1.25, and 1.16, respectively (Table S1). Five variables were common to all models (dominant diameter, stand age, tree number per hectare, Gini coefficient, and mean tree height) and consistently ranked above all other factors. Beyond these shared variables, the pure Chinese fir also included mean annual precipitation, mean temperature of the warmest month, and soil organic carbon. The Chinese fir–broadleaf stands additionally included the mean temperature of the warmest month. Moreover, and the Chinese fir–conifer stands additionally included soil organic carbon and bulk density. Ten‐fold cross‐validation revealed that the productivity models for all stand types performed well in terms of *R* ^2^, MAE, and RMSE. The differences between the predicted and observed values were small. Details about the optimal feature combinations and evaluation results are presented in Figure 5b.

### 3.3 Pathways of Influencing Factors on Productivity

**FIGURE 6:**
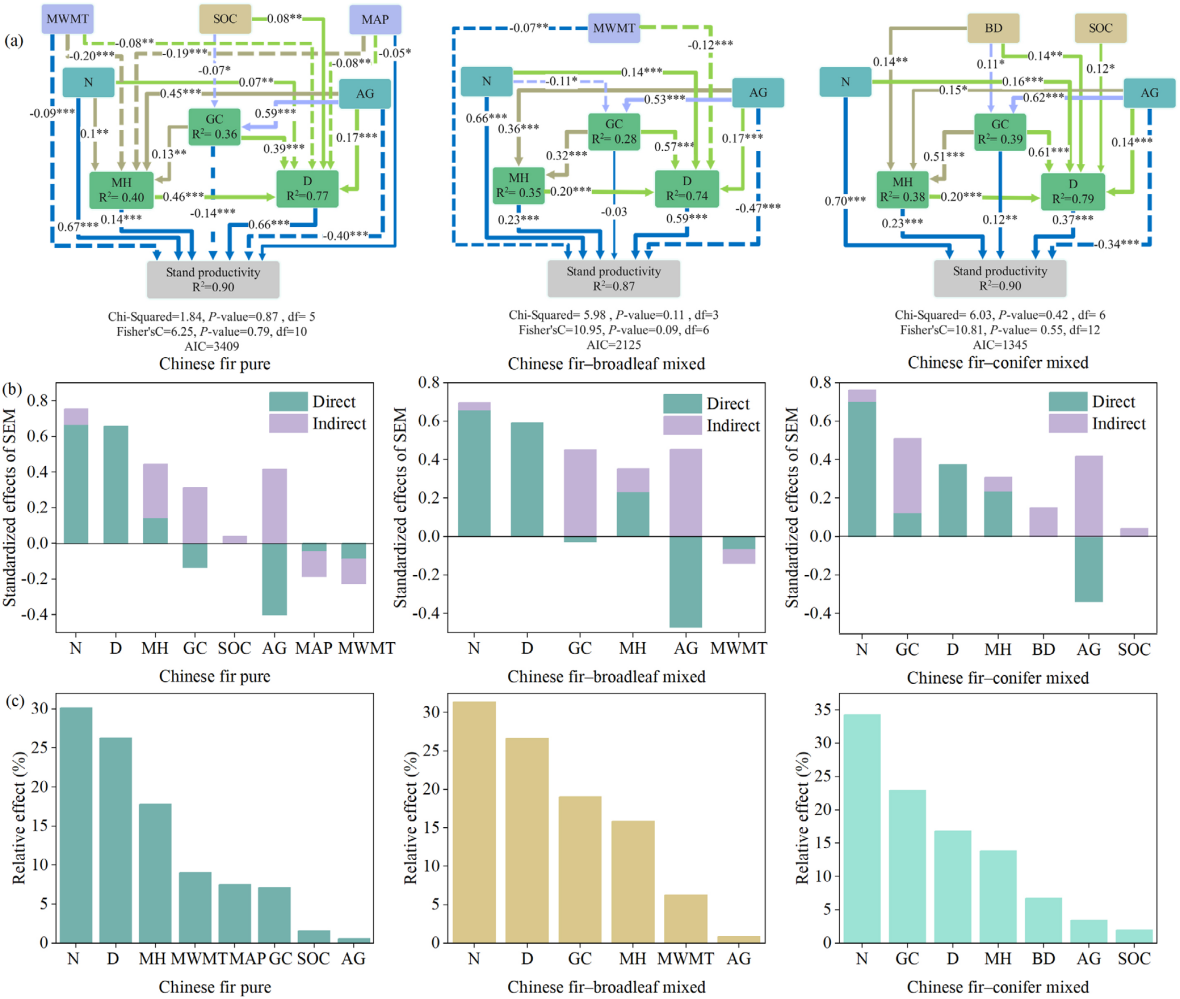
Structural Equation Models of different Chinese fir stand types. (a) Structural equation model; (b) direct and indirect effects; (c) relative effects. Different colors represent different sub‐models. Solid and dashed lines indicate positive and negative paths, respectively. The numbers next to the paths are standardized path coefficients, with asterisks indicating significant paths (*p < 0.05, **p < 0.01, ***p < 0.001). *R* ^2^ represents the proportion of variance explained. The sum of direct and indirect effects equals the total effect. Relative effects are ranked according to the contribution of each factor to the total effect. AG, stand age; BD, bulk density; *D*, dominant tree diameter; GC, Gini coefficient; MAP, mean annual precipitation; MH, mean tree height; MWMT, mean warmest month temperature; *N*, tree number per hectare; SOC, soil organic carbon.

We examined variable collinearity and confirmed the absence of multicollinearity (Table S2); bivariate relationships are illustrated in Figure S2. To explore the pathways through which the main factors influence productivity, including both direct and indirect effects, we first constructed a full model with all candidate paths (Figure S3). Based on this, we removed non‐significant paths as much as possible while ensuring acceptable overall model fit according to Fisher's C and Chi‐square tests (*p* > 0.05), resulting in the simplified model with the lowest AIC. Consequently, we constructed SEMs for the three Chinese fir stand types (Figure 6). Figure 6a illustrates the influence pathways among variables and the model fit (Fisher's *C*, Chi‐square, AIC, and *R* ^2^ values). The SEMs explained variations in productivity with *R* ^2^ values of 0.90, 0.87, and 0.90 for pure Chinese fir, Chinese fir–broadleaf mixed, and Chinese fir–conifer mixed stands, respectively. These results indicate that the constructed SEMs are a good fit for the data. Although there were significant pathways among the influencing factors, the pathways and relative impacts on productivity varied across the different stand types. The direct and indirect effect coefficients of each factor are presented in Figure 6b, and their relative effects are presented in Figure 6c.

As presented in Figure 6a, the impact of the influence pathways, effect standardized coefficient, and significance levels among stand factors (tree number per hectare, dominant diameter, stand age, Gini coefficient, and mean tree height) on stand productivity varied. The total effects of stand factors on productivity were mostly positive (Figure 6b). Tree number per hectare exhibited the strongest total effect on productivity across all stand types (Chinese fir pure = 0.75, Chinese fir–broadleaf mixed = 0.70, Chinese fir–conifer mixed = 0.76). In contrast, stand age demonstrated the lowest total effect, with a significant negative direct effect (Chinese fir pure = −0.40, Chinese fir–broadleaf mixed = −0.47, Chinese fir–conifer mixed = −0.34). However, stand age also exerted an indirect positive effect through its influence on stand structural attributes, including dominant diameter, mean tree height, and Gini coefficient (Chinese fir pure = 0.42, Chinese fir–broadleaf mixed = 0.45, Chinese fir–conifer mixed = 0.42).

As illustrated in Figure 6c, among the stand factors, tree number per hectare had the greatest relative effect on stand productivity across all stand types, followed by either dominant diameter or the Gini coefficient, whereas stand age exhibited the smallest effect. The relative contributions of the Gini coefficient to productivity were 7.11%, 19.03%, and 22.93% for pure Chinese fir, Chinese fir–broadleaf mixed, and Chinese fir–conifer mixed stands, respectively. As presented in Figure 6a, the dominant diameter was most strongly influenced by mean tree height in pure stands, whereas the diameter was most strongly influenced by the Gini coefficient in both mixed stand types. These results indicate that the Gini coefficient has a greater impact on productivity in mixed stands than in pure stands.

Climatic and site factors affected productivity differently across the three Chinese fir stand types. As depicted in Figure 6a,c, the mean temperature of the warmest month had a stronger influence on productivity in pure Chinese fir stands (relative influence = 0.09%) and more complex pathways than in Chinese fir–broadleaf mixed stands (relative influence = 0.06%). Similarly, soil organic carbon exhibited more influence pathways in pure stands (SOC → D, SOC → GC) than in Chinese fir–conifer mixed stands (SOC → D). These results indicate that pure Chinese fir stands were more sensitive to climatic and site factors, reflecting a stronger response to environmental changes.

## 4 Discussion

### 4.1 Comparison of the Productivity of Chinese Fir Pure and Mixed Stands

Mixed forests often exhibit an “overyielding” effect compared to pure forests. For example, Pretzsch and Forrester (2017) examined permanent plots and forest inventory data and found that the overyielding of mixed forests in temperate and boreal zones reached 10%–30%. Similarly, Jactel et al. (2018) reported that productivity was 15% higher on average in mixed forests than in monocultures across five continents. However, some studies found no correlation between species diversity and productivity (Szwagrzyk and Gazda 2007). Consistently, our study revealed no significant overyielding effects in mixed stands. The productivity in pure Chinese fir stands was higher than that in mixed stands, with Chinese fir–broadleaf mixed stands surpassing Chinese fir–conifer mixed stands. Cao and Chen (2000) reported that 15‐year‐old pure Chinese fir stands in Fujian, China, were more productive than Chinese fir–Masson pine and Chinese fir–Masson pine–*Schima* mixed stands. Similarly, Zhou et al. (2024) reported that the volume of 20‐year‐old pure Chinese fir stands was 9.66% higher than that of Chinese fir–*Elaeocarpus sylvestris* mixed stands. Conversely, some studies have indicated increased productivity in mixed stands; for example, a study in Sichuan, China, demonstrated that the productivity of Chinese fir–*Cryptomeria fortunei* mixed stands exhibited 39.5% higher than that of pure Chinese fir stands (Yang et al. 2023). Additionally, Jiang et al. (2023) reported that productivity was higher in 28‐year‐old Chinese fir–*Mytilaria laosensis* and Chinese fir–*Michelia macclurei* mixed stands than in pure Chinese fir stands. Despite these inconsistent results on the productivity of pure and mixed Chinese fir stands, our study demonstrated significant differences among stand types, supporting the hypothesis that productivity varies with stand type.

Differences in functional traits, niches, and other characteristics among tree species lead to varying competition and complementarity effects. In addition, factors such as region, site quality, and mixed‐species configurations can result in productivity differences between pure and mixed stands. In high‐quality sites, pure stands tend to achieve higher productivity than mixed stands (Toïgo et al. 2015). Moreover, potential neutral or negative interactions among species may result in lower productivity in coniferous mixed stands than in monospecific forests (Aguirre et al. 2019). However, strong interspecific complementarity often leads to high productivity in conifer–broadleaf mixed stands (Feng et al. 2022). Chinese fir is a native species widely planted in the subtropical regions of southern China. Owing to their ease of management, pure stands have long been the dominant plantation type. In the present study area, pure stands are primarily managed for timber production, often through intensive silvicultural practices. This management approach may partly explain why the productivity of pure Chinese fir stands exceeded that of mixed stands. Another possible explanation lies in the differences in the magnitude and pathways through which stand, site, and climatic factors influence productivity in pure versus mixed stands. In this study, the same set of 20 variables was tested across different stand types, and their importance was ranked based on %IncMSE from the RF model revealing that stand factors are highly important for predicting productivity. Therefore, we further applied SEM to test Hypothesis 2, which examines how different influencing factors affect stand productivity through stand structure.

### 4.2 Factors Influencing Chinese Fir Stand Productivity

Our results indicate that climate, site, and stand factors affect productivity directly or indirectly through stand structure, and that their pathways and relative importance differ among stand types. Specifically, tree number per hectare, dominant tree diameter, mean tree height, Gini coefficient, and stand age influence productivity across pure Chinese fir, Chinese fir–broadleaf mixed, and Chinese fir–conifer mixed stands. Among these variables, tree number per hectare, mean tree height, and dominant diameter consistently exerted strong positive effects on productivity in all stand types. In contrast, stand age exerted a negative direct effect on productivity but exhibited consistently positive indirect effects through stand structure. Some studies have reported a positive relationship between stand age and productivity (Ouyang et al. 2019; Xiang et al. 2022), whereas others have observed a negative effect in Chinese fir stands (Wang et al. 2021). These discrepancies may be attributed to differences in region, stand type, or developmental stage. In the present study, the plots were generally young to mid‐aged, with natural self‐thinning occurring following canopy closure. Self‐thinning and growth deceleration after canopy closure can lead to a direct decline in productivity with increasing age (Yang et al. 2019). However, our path analysis revealed that stand age positively influenced dominant diameter, Gini coefficient, and mean tree height (Figure 6a), indicating that structural improvement associated with aging may enhance productivity through indirect pathways. Notably, similar findings were reported by Matsuo et al. (2025).

Stand density and productivity typically follow a unimodal relationship where productivity may decline once density exceeds a certain threshold due to intensified resource competition (Feng et al. 2022). However, the stand density in our study plots was generally low, particularly in mixed forests, where the potential of maximum density to enhance productivity was not fully realized (Morin et al. 2025). Large trees have a strong capacity for resource acquisition and utilization and can maintain high growth rates throughout their life cycle (Stephenson et al. 2014). According to long‐term observations, the contribution of large trees to biomass accumulation exceeds the losses caused by stand mortality (Shi et al. 2025). Therefore, promoting dominant trees through targeted management is an effective strategy for improving stand productivity. Moreover, mean tree height positively influences productivity (Pan et al. 2023), as greater height indicates higher growth potential (Duan et al. 2022). Our results are consistent with these findings.

The Gini coefficient, which reflects size inequality among trees, exhibited varying effects on productivity across the three stand types. As illustrated in Figure 6c, its relative effect on productivity was greater in mixed stands than in pure stands, although total effects remained consistently positive across all types (Figure 6b). Similarly, Wang et al. (2021) reported a positive correlation between the Gini coefficient and productivity in Chinese fir plantations. Compared with pure stands, mixed stands exhibit enhanced structural complexity expressed through crown heterogeneity, light stratification, and functional trait diversity. Such complexity can enhance resource‐use complementarity and thereby improve productivity (Ray et al. 2023). In mixed stands, differences in leaf morphology (needleleaf vs. broadleaf) and leaf lifespan (deciduous vs. evergreen) among species enhance spatiotemporal complementarity in resource use, thereby improving light‐use efficiency and stand productivity (Feng et al. 2022). In our study, both mixed Chinese fir stands exhibited higher mean Gini coefficients than pure stands (Table 1), and productivity increased with increasing Gini coefficients in the mixed stands (Figure 3d). Structural heterogeneity in mixed forests tends to become more pronounced in later successional stages (Pretzsch and Schütze 2016), further enhancing their long‐term productivity potential. Jing et al. (2021) also observed more differentiated root distributions in mixed stands, which facilitates deeper and more efficient use of soil resources compared to pure stands. Therefore, mixed stands demonstrated enhanced productivity potential during later stages of stand development. In pure stands, owing to their relatively homogeneous structure, the Gini coefficient exhibits a threshold effect, with only moderate size differentiation enhancing productivity (Pretzsch et al. 2024). Our findings also indicated that productivity in pure Chinese fir stands first increased and then decreased with rising Gini values (Figure 3d). Therefore, structural optimization through appropriate enrichment planting of broadleaf species could gradually transform pure stands into mixed stands, which would be beneficial for maintaining sustainable productivity.

In addition to the common stand variables, the productivity in the three stand types was influenced by climatic and site factors; however, their effects differed to some extent among the stand types. In pure Chinese fir stands, additional main factors included the mean annual precipitation, the mean temperature of the warmest month, and soil organic carbon. In Chinese fir–broadleaf mixed stands, productivity was further affected by the mean temperature of the warmest month, whereas in Chinese fir–conifer mixed stands, soil organic carbon and bulk density played important roles.

Among climatic variables, both pure Chinese fir and Chinese fir–broadleaf mixed stands were negatively affected by the mean temperature of the warmest month, with a stronger effect observed in pure stands (Figure 6b). Liu et al. (2024) reported that an increase in mean temperature in the warmest month significantly raised the risk of mortality in Chinese fir forests in Jiangxi Province, whereas conifer–broadleaf mixed forests exhibited stronger recovery and drought resistance than pure stands (Pardos et al. 2021). In contrast, in our study, Chinese fir–conifer mixed stands exhibited decreased sensitivity to climate variation and were instead more influenced by site (Figure 4). This may be because in our study area, Chinese fir–conifer mixed stands were generally established or naturally developed on relatively poor‐quality sites, where *Pinus massoniana* —a native pioneer species commonly used for afforestation and greening—was the main mixed species. Some studies have suggested that the effects of climatic factors are often weakened or overshadowed under poor site conditions. For example, Huber et al. (2021) found that in Swiss forest inventory plots, stand growth was less sensitive to climatic factors under poor site conditions such as low soil quality. In semiarid Mediterranean regions, even abundant precipitation may not promote vegetation recovery if site conditions are poor, reflecting an antagonistic interaction between climate and site factors (García‐Fayos and Bochet 2009). Similarly, Wang, Zhang, et al. (2022) reported that site conditions dominated the succession of plant functional traits during stages of soil impoverishment in karst areas, potentially masking the effects of climate factors. Overall, the productivity of pure Chinese fir stands was more strongly affected by climatic and site factors compared to that of mixed stands. Moreover, mixed‐species forests exhibit greater ecological stability and adaptability than pure stands. For example, forests with higher species diversity can enhance litter decomposition and nutrient cycling by increasing the diversity of litter inputs and the complexity of decomposer communities, contributing to maintaining site productivity (Luan et al. 2024). Under ongoing climate warming and increasingly frequent extreme weather events, large‐scale mortality in pure conifer plantations has been widely reported (Mcdowell et al. 2016). Converting pure conifer plantations into conifer–broadleaf mixed forests through positive succession can enhance forest ecosystem stability and resilience to climate change while also improving site quality, productivity, and carbon storage (Liu et al. 2018). Overall, mixed forests exhibit stronger resilience to climate change than pure stands, with Chinese fir–broadleaf mixed stands exhibiting particular advantages in maintaining site productivity. Although this study provides regional evidence on the productivity of different Chinese fir stands, data on microclimate, soil, and light conditions were limited. Future research should focus on collecting experimental control data of plots to address these gaps and further investigate species‐level growth and environmental impacts.

## 5 Conclusion

This study, conducted in subtropical China, revealed significant productivity differences among pure Chinese fir, Chinese fir–broadleaf mixed, and Chinese fir–conifer mixed stands (*p* < 0.05). Climate, site, and stand factors influenced productivity directly or indirectly through stand structure; however, their main influencing factors varied across stand types. Tree density, dominant tree diameter, Gini coefficient, mean tree height, and stand age were common key factors, with tree density exerting the strongest effect. Size inequality exhibited a strong positive effect on mixed stands, whereas the productivity of pure stands was more sensitive to climatic and site conditions. These findings highlight the key role of stand structure in regulating productivity, suggesting that optimizing tree density and structural heterogeneity can enhance both productivity and stability. Moreover, pure stands were more sensitive to climatic and site conditions, while mixed stands were more influenced by structural heterogeneity. Over longer rotations, mixed stands hold great potential for productivity improvement and stability. However, future long‐term experiments are needed to confirm the causal effects of density regulation and mixed‐species management.

## Author Contributions

**Yang Guo:** conceptualization (equal), formal analysis (equal), methodology (equal), software (equal), validation (equal), writing – original draft (equal), writing – review and editing (equal). **Xunzhi Ouyang:** data curation (equal), funding acquisition (equal), methodology (equal), project administration (equal), resources (equal), supervision (equal), writing – original draft (equal). **Ping Pan:** conceptualization (equal), funding acquisition (equal), supervision (equal). **Jianfeng Li:** conceptualization (equal), methodology (equal). **Jun Liu:** conceptualization (equal), data curation (equal).

## Funding

This work was supported by the National Natural Science Foundation of China (grant nos. 32360389 and 32260392) and the Natural Science Foundation of Jiangxi Province (20232BAB215048).

## Conflicts of Interest

The authors declare no conflicts of interest.

## Supporting information


**Appendix S1:** ece372750‐sup‐0001‐AppendixS1.docx.

## Data Availability

The forest inventory data supporting the results of this study come from the 2019 National Second‐Class Forest Resource Survey of China. Due to policy restrictions, these raw data are not publicly available and can only be accessed upon approval from the relevant administrative authorities. Climate data were obtained from the publicly available database ClimateAP v3.10, and soil data were sourced from SoilGrids (https://soilgrids.org/). All simulated data, codes, and Supporting Information figures generated in this study have been deposited in Figshare and are openly available at the following link: https://doi.org/10.6084/m9.figshare.30189961.
