# Supplementary material for: Stand Structure and Type Drive Productivity in Chinese Fir Forests: Comparison of Pure and Mixed Stands
Source: Ecol Evol. 2026 Jan 11;16(1):e72750. doi: 10.1002/ece3.72750 (PMC12790873; doi:10.1002/ece3.72750)
Supplement: Supplementary file 1 — Appendix S1: ece372750‐sup‐0001‐AppendixS1.docx. [file ECE3-16-e72750-s001.docx]

Supporting Information for:

**Stand structure and type drive productivity in Chinese fir forests: Comparison of pure and mixed stands**

**
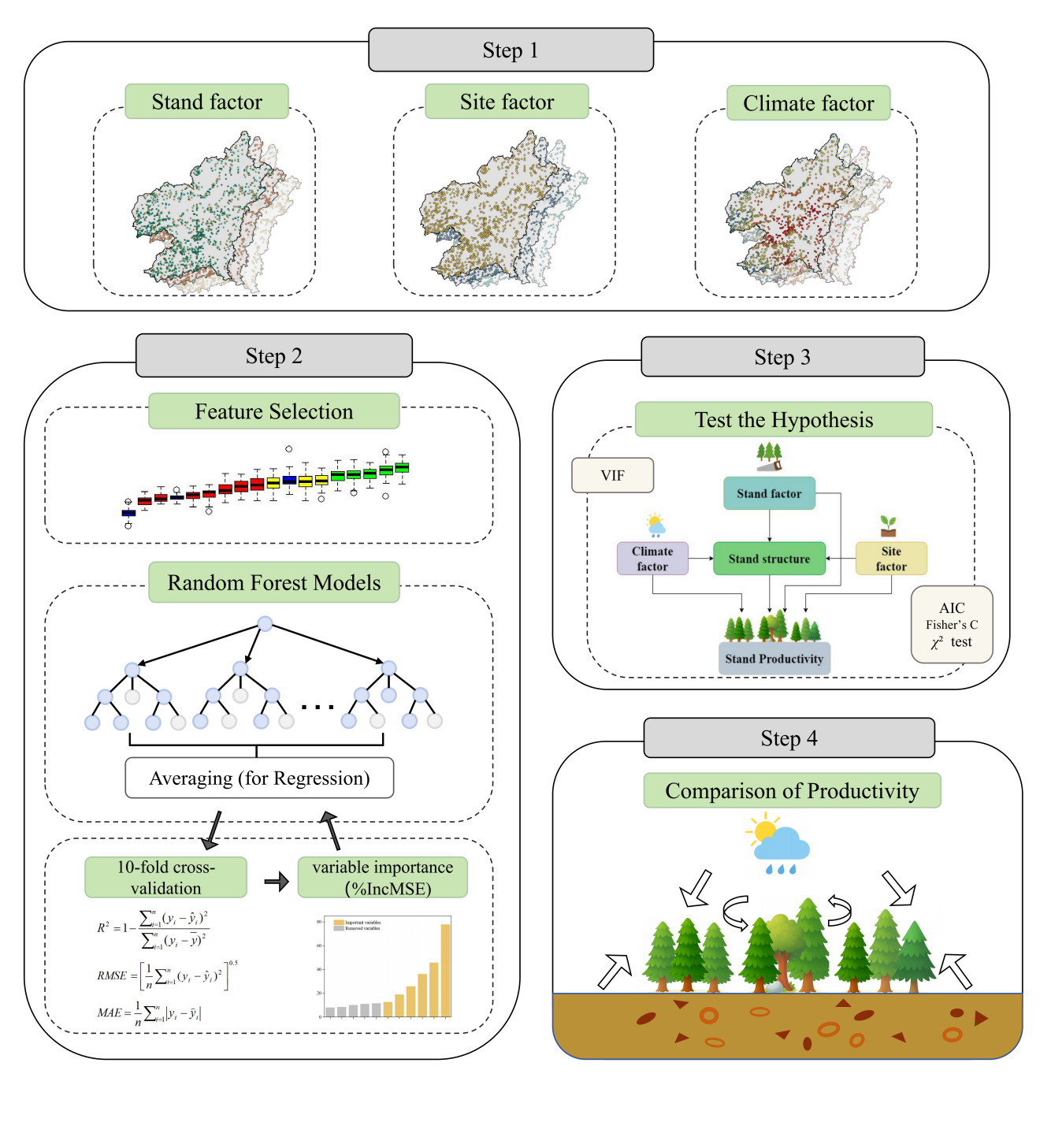
**

**FIGURE S1** Stepwise analytical framework for comparing stand productivity mechanisms in Chinese fir forests. The framework consists of four main steps: (1) data preparation of stand, site, and climate factors; (2) feature selection and random forest modeling; (3) hypothesis testing using structural equation modeling (SEM); and (4) comparison of stand productivity mechanisms among different stand types based on SEM results.

**TABLE S1** Performance of random forest models

| **Stand type** | **Model No.** | **Variables** | **10-fold cross-validation** | | | |
| --- | --- | --- | --- | --- | --- | --- |
|  |  |  | **mtry** | **R2** | **RMSE** | **MAE** |
| Chinese fir pure | RF1 | N+D+AG+MH+GC+SOC+MWMT+MAP+ELE+TN+MAT+RH+BD+MCMT | 12 | 0.768 | 2.298 | 1.611 |
|  | RF2 | N+D+AG+MH+GC+SOC+MWMT+MAP+ELE+TN+MAT+RH+BD | 12 | 0.781 | 2.234 | 1.547 |
|  | RF3 | N+D+AG+MH+GC+SOC+MWMT+MAP+ELE+TN+MAT+RH | 11 | 0.787 | 2.211 | 1.55 |
|  | RF4 | N+D+AG+MH+GC+SOC+MWMT+MAP+ELE+TN+MAT | 9 | 0.782 | 2.244 | 1.551 |
|  | RF5 | N+D+AG+MH+GC+SOC+MWMT+MAP+ELE+TN | 7 | 0.788 | 2.213 | 1.547 |
|  | RF6 | N+D+AG+MH+GC+SOC+MWMT+MAP+ELE | 6 | 0.798 | 2.19 | 1.511 |
|  | RF7 | N+D+AG+MH+GC+SOC+MWMT+MAP | 7 | 0.808 | 2.142 | 1.494 |
|  | RF8 | N+D+AG+MH+GC+SOC+MWMT | 7 | 0.795 | 2.163 | 1.492 |
|  | RF9 | N+D+AG+MH+GC+SOC | 4 | 0.803 | 2.161 | 1.473 |
|  | RF10 | N+D+AG+MH+GC | 4 | 0.81 | 2.143 | 1.452 |
|  | RF11 | N+D+AG+MH | 3 | 0.782 | 2.185 | 1.54 |
|  | RF12 | N+D+AG | 2 | 0.754 | 2.331 | 1.676 |
|  | RF13 | N+D | 1 | 0.529 | 3.256 | 2.361 |
|  | RF14 | N | 1 | 0.331 | 3.92 | 2.994 |
| Chinese fir–broadleaf mixed | RF15 | N+D+AG+MH+GC+MWMT+MCMT+MAT+ELE+ST+SOC | 10 | 0.762 | 1.306 | 0.98 |
|  | RF16 | N+D+AG+MH+GC+MWMT+MCMT+MAT+ELE+ST | 7 | 0.762 | 1.327 | 0.988 |
|  | RF17 | N+D+AG+MH+GC+MWMT+MCMT+MAT+ELE | 8 | 0.752 | 1.316 | 0.983 |
|  | RF18 | N+D+AG+MH+GC+MWMT+MCMT+MAT | 6 | 0.773 | 1.289 | 0.965 |
|  | RF19 | N+D+AG+MH+GC+MWMT+MCMT | 6 | 0.763 | 1.301 | 0.97 |
|  | RF20 | N+D+AG+MH+GC+MWMT | 5 | 0.78 | 1.253 | 0.93 |
|  | RF21 | N+D+AG+MH+GC | 4 | 0.764 | 1.295 | 0.96 |
|  | RF22 | N+D+AG+MH | 3 | 0.761 | 1.299 | 0.963 |
|  | RF23 | N+D+AG | 2 | 0.722 | 1.388 | 1.023 |
|  | RF24 | N+D | 1 | 0.578 | 1.707 | 1.285 |
|  | RF25 | N | 1 | 0.302 | 2.287 | 1.773 |
| Chinese fir–conifer mixed | RF26 | N+D+MH+AG+GC+SOC+BD+TN+RH+ST | 10 | 0.783 | 1.289 | 0.913 |
|  | RF27 | N+D+MH+AG+GC+SOC+BD+TN+RH | 9 | 0.761 | 1.266 | 0.887 |
|  | RF28 | N+D+MH+AG+GC+SOC+BD+TN | 6 | 0.784 | 1.211 | 0.855 |
|  | RF29 | N+D+MH+AG+GC+SOC+BD | 4 | 0.81 | 1.163 | 0.841 |
|  | RF30 | N+D+MH+AG+GC+SOC | 4 | 0.78 | 1.218 | 0.847 |
|  | RF31 | N+D+MH+AG+GC | 3 | 0.792 | 1.226 | 0.835 |
|  | RF32 | N+D+MH+AG | 3 | 0.774 | 1.201 | 0.833 |
|  | RF33 | N+D+MH | 3 | 0.687 | 1.43 | 0.989 |
|  | RF34 | N+D | 1 | 0.632 | 1.555 | 1.121 |
|  | RF35 | N | 1 | 0.5 | 1.833 | 1.375 |

Note: This table presents the performance of random forest models with different variable combinations across three Chinese fir stand types. All models were evaluated using 10-fold cross-validation, ntree=1000 and the optimal value of mtry was selected through parameter tuning. RF7 (pure stand), RF20 (Chinese fir–broadleaf mixed stand), and RF29 (Chinese fir–conifer mixed stand) were the optimal models. The corresponding variable combinations can be considered key factors influencing stand productivity.

**TABLE S2** Variance inflation factor analysis for multicollinearity diagnosis

| **Variables** | **Variance inflation factor** | | |
| --- | --- | --- | --- |
|  | **Chinese fir pure** | **Chinese fir–broadleaf mixed** | **Chinese fir–conifer mixed** |
| D | 4.36 | 3.78 | 4.78 |
| GC | 2.26 | 2.76 | 3.87 |
| AG | 2.23 | 1.73 | 1.80 |
| MH | 2.60 | 1.72 | 1.85 |
| N | 1.04 | 1.08 | 1.16 |
| MWMT | 2.02 | 1.13 | - |
| MAP | 1.94 | - | - |
| SOC | 1.52 | - | 2.87 |
| BD | - | - | 2.96 |


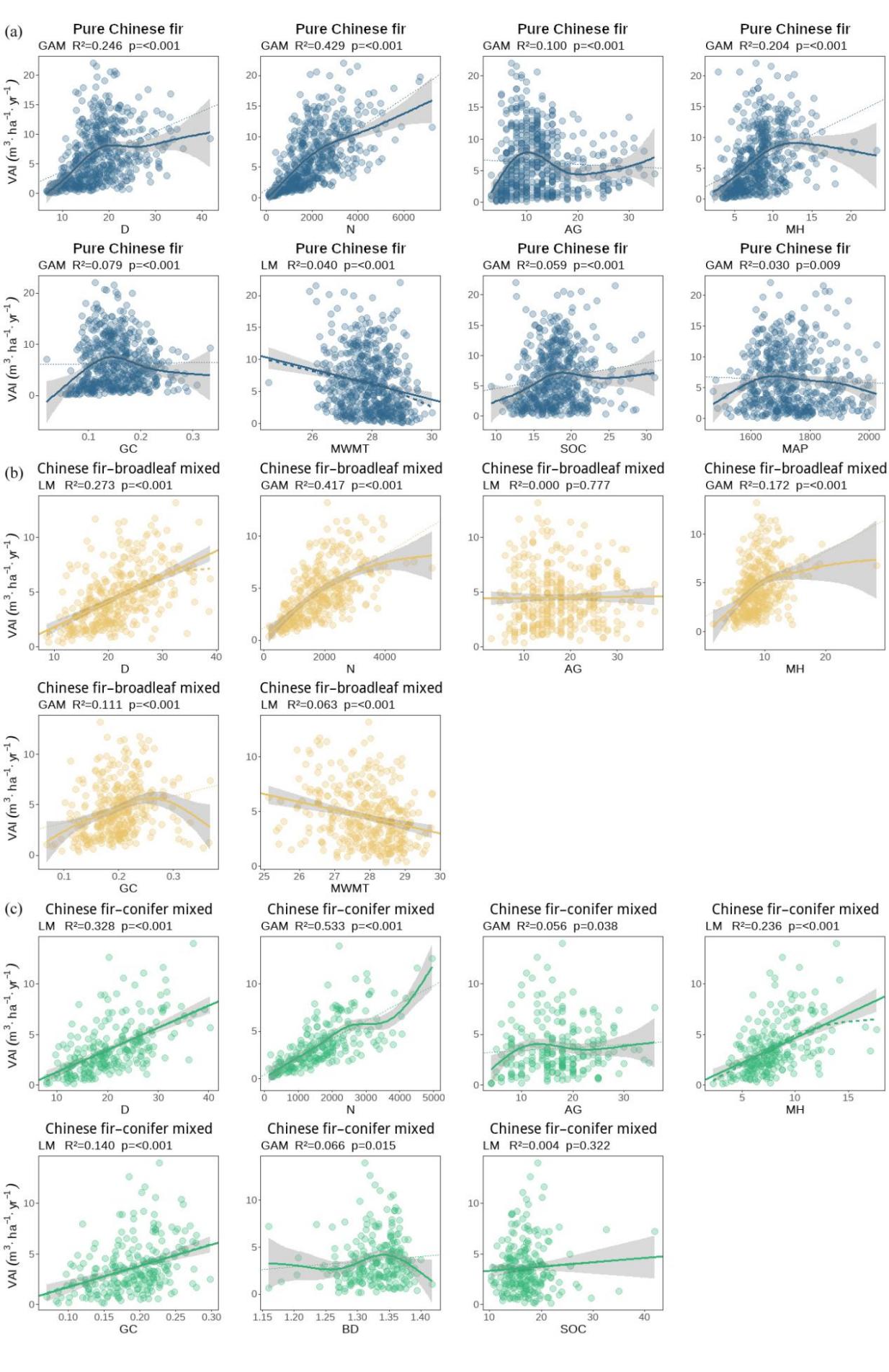


**FIGURE S2** Relationships between productivity and main factors under different stand types. For each variable, both ordinary least squares (OLS) and generalized additive models (GAM) were fitted; GAM was adopted when the nonlinearity test was significant (*p*<0.05) andΔAIC≥2, otherwise OLS was used.


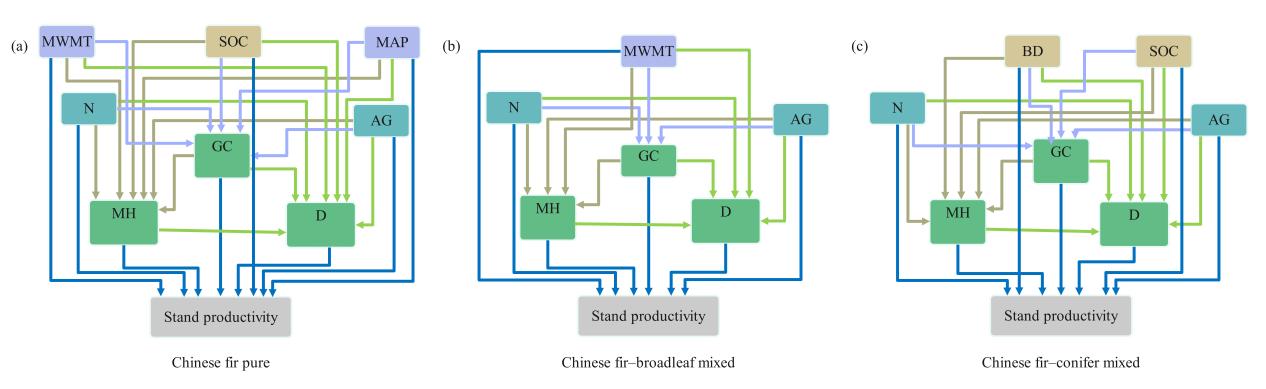


**FIGURE S3** Saturated structural equation models for different stand types (a–c). Climate and site variables and management-related stand variables are proposed to influence productivity either directly or indirectly through key stand structural factors. MAP: mean annual precipitation; MWMT: mean warmest month temperature; SOC: soil organic carbon; BD: bulk density; N: stand density; AG: stand age; D: dominant tree diameter; MH: mean tree height; GC: Gini coefficient.
